# Sterols from the Green Alga *Ulva australis*

**DOI:** 10.3390/md15100299

**Published:** 2017-09-28

**Authors:** Guo-Liang Li, Wei-Jie Guo, Guang-Bao Wang, Rong-Rong Wang, Yu-Xue Hou, Kun Liu, Yang Liu, Wei Wang

**Affiliations:** Department of Natural Medicine and Pharmacognosy, School of Pharmacy, Qingdao University, Qingdao 266071, China; qdguoliangli@163.com (G.-L.L.); wfenterance@163.com (W.-J.G.); qddxgbwang@163.com (G.-B.W.); rrwang2012@163.com (R.-R.W.); hyx19931008@163.com (Y.-X.H.); kunliu62@126.com (K.L.); buckuper@163.com (Y.L.)

**Keywords:** Chlorophyta, *Ulvaaustralis*, 5,28-stigmastadiene-3β,24-diol-7-one epimers, 24-vinylcholesta-3β,5α,6β,24-tetraol

## Abstract

Three new sterols, (24*R*)-5,28-stigmastadiene-3β,24-diol-7-one (**1**), (24*S*)-5,28-stigmastadiene-3β,24-diol-7-one (**2**), and 24*R* and 24*S*-vinylcholesta-3β,5α,6β,24-tetraol (**3**), together with three known sterols (**4**–**6**) were isolated from the green alga *Ulva australis*. The structures of the new compounds (**1**–**3**) were elucidated through 1D and 2D nuclear magnetic resonance spectroscopy as well as mass spectrometry. Compounds **4**–**6** were identified as isofucoterol (**4**), 24*R*,28*S* and 24*S*,28*R*-epoxy-24-ethylcholesterol (**5**), and (24*S*)-stigmastadiene-3β,24-diol (**6**) on the basis of spectroscopic data analyses and comparison with those reported in the literature. Compounds **4**–**6** were isolated from *U*. *australis* for the first time. These compounds, together with the previously isolated secondary metabolites of this alga, were investigated for their inhibitory effects on human recombinant aldose reductase in vitro. Of the compounds, 24*R*,28*S* and 24*S*,28*R*-epoxy-24-ethylcholesterol (**5**), 1-*O*-palmitoyl-3-*O*-(6′-sulfo-α-d-quinovopyranosyl) glycerol, (2*S*)-1-*O*-palmitoyl-3-*O*-[α-d-galactopyranosyl(1→2)β-d-galactopyranosyl] glycerol, 4-hydroxybenzoic acid, 4-hydroxyphenylacetic acid, and 8-hydroxy-(6*E*)-octenoic acid weakly inhibited the enzyme, while the three new sterols, **1**–**3,** were almost inactive.

## 1. Introduction

**Figure 1:**
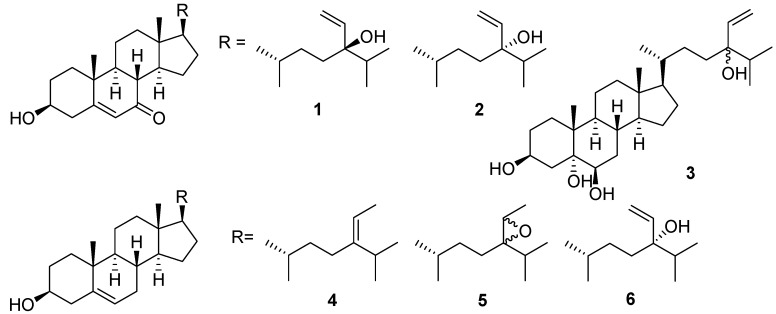
Structures of compounds **1**–**6**.

Marine organisms have recently received much attention in the search for structurally interesting compounds with a wide range of pharmacological activities to develop new medicines or health foods [1,2,3,4,5]. Approximately 8000 species of marine algae have been identified and grouped into different classes, including brown algae (Phaeophyta), red algae (Rhodophyta), and green algae (Chlorophyta). *Ulva australis* Areschoug is a green alga belonging to the family Ulvaceae and is widely distributed along the coasts of the Yellow Sea and the Bo Sea of China. As an edible seaweed, it contains high nutritional value minerals, vitamins, and noncaloric dietary fibers [6]. *Ulva australis* is consumed by local inhabitants of Asia [7] and has been authorized for human utilization by French authorities [8]. In addition to edibility, the decoction of this alga has been accepted as a natural traditional medicine for hyperlipidemia, sunstroke, and urinary diseases [9]. The chemical and pharmacological studies of *U*. *australis* available in the literature are mostly concerned with the algal polysaccharides. These sulfated polysaccharides have been described to possess diverse biological activities, such as antihyperlipidemic [10], antioxidant [11], antiviral [12], immunomodulatory [13], and anti-radiation activities [14]. However, small-molecule chemical components of *U*. *australis* and structure identification have only received limited attention [15,16,17]. It has been recently reported that 3-hydroxy-4,7-megastigmadien-9-one, isolated from this alga, attenuates lipopolysaccharide-induced and TLR9-mediated inflammatory responses by downregulating mitogen-activated protein kinase and NF-κB pathways [7,18]. In our search for aldose reductase inhibitors from marine algae, we found that bromophenols isolated from the red alga *Symphyocladia latiuscula* exhibited significant human recombinant aldose reductase inhibitory activity [19]. The extract from *U*. *australis* exhibited weak human recombinant aldose reductase inhibitory activity, which is in agreement with the previous report [20]. Our continued interest in discovering new secondary metabolites from marine algae led us to isolate three new sterols, (24*R*)-5,28-stigmastadiene-3β,24-diol-7-one (**1**), (24*S*)-5,28-stigmastadiene-3β,24-diol-7-one (**2**), and 24*R* and 24*S*-vinylcholesta-3β,5α,6β,24-tetraol (**3**), together with three known sterols, isofucoterol (**4**), 24*R*,28*S* and 24*S*,28*R*-epoxy-24-ethylcholesterol (**5**), and (24*S*)-5,28-stigmastadiene-3β,24-diol (**6**), from the green alga *U*. *australis* (Figure 1). The structures of these compounds were identified by NMR spectroscopy, mass spectrometry, and compared with those reported in the literature. The configurations of compounds **1** and **2** were determined on the basis of the chemical shift differences between H-27 and H-26 and between H-27 and H-21. Herein, we also report on the evaluation of the human recombinant aldose reductase inhibitory activities of the compounds isolated by us from this alga.

## 2. Results and Discussion

**Figure 2:**
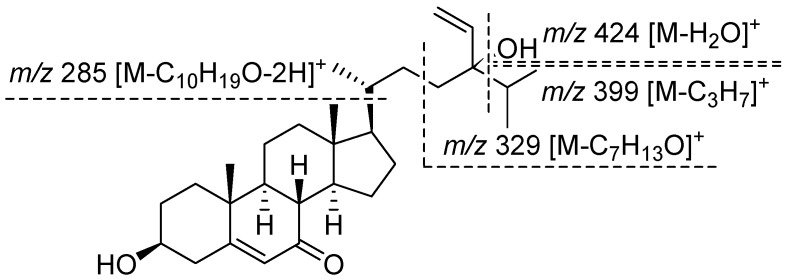
EI-MS fragments of compound **1**.

Compound **1** was isolated as a white amorphous solid. The molecular formula was determined to be C_29_H_46_O_3_ by HR-EI-MS at *m*/*z* 442.3445 (calcd. for C_29_H_46_O_3_ 442.3447). The sterol nature of this compound was deduced from a combination of ^13^C NMR (Table 1) and distortionless enhancement by polarization transfer spectra. The ^13^C NMR spectrum of compound **1** showed 29 resonances, which were assigned to five methyl groups (δ_C_ 11.9 (C-18), 16.4 (C-26), 17.3 (C-19), 17.5 (C-27), and 18.9 (C-21)), nine sp^3^ methylene carbons (δ_C_ 21.2 (C-11), 26.2 (C-15), 29.0 (C-22), 29.6 (C-16), 31.1 (C-2), 34.8 (C-23), 36.3 (C-1), 38.6 (C-12), and 41.7 (C-4)), one sp^2^ methylene carbon (δ_C_ 112.7 (C-29)), six sp^3^ methine carbons (δ_C_ 35.8 (C-25), 36.0 (C-20), 45.3 (C-8), 49.8 (C-14), 49.8 (C-9), and 54.4 (C-17)), one oxygenated sp^3^ methine carbons (δ_C_ 70.4 (C-3)), two sp^2^ methine carbons (δ_C_ 125.8 (C-6) and 142.1 (C-28)), two sp^3^ quaternary carbons (δ_C_ 38.2 (C-10) and 43.0 (C-13)), one oxygenated sp^3^ quaternary carbon (δ_C_ 77.5 (C-24)), one sp^2^ quaternary carbon (δ_C_ 164.6 (s, C-5)), and one ketone carbonyl carbon (δ_C_ 201.6 (C-7)). The ^1^H NMR spectrum of compound **1** had resonances corresponding to two tertiary methyl groups (δ_H_ 0.68 (s, H-18) and 1.20 (s, H-19)), three secondary methyl groups (δ_H_ 0.87 (d, *J* = 7.2 Hz, H-26), 0.89 (d, *J* = 8.0 Hz, H-27), and 0.93 (3H, d, *J* = 6.4 Hz, H-21)), an oxymethine proton (δ_H_ 3.67 (m, H-3)), and an olefinic proton (δ_H_ 5.69 (d, *J* = 1.6 Hz, H-6)). The ^1^H NMR spectrum exhibited an ABX system at δ_H_ 5.14 (1H, dd, *J* = 10.8, 1.6 Hz, H-29), 5.20 (1H, dd, *J* = 17.6, 1.6 Hz, H-29), and 5.81 (1H, dd, *J* = 17.6, 10.8, H-28), due to the presence of a vinyl group attached to a tertiary carbon. These data, along with mass fragments at *m*/*z* 424 [M − H_2_O]^+^, 399 [M − C_3_H_7_]^+^, 381 [M − C_3_H_7_ − H_2_O]^+^, 329 [M − C_7_H_13_O]^+^, and 285 [M − C_10_H_19_O − 2H]^+^ (Figure 2) suggested that compound **1** possessed a 24-hydroxy-24-vinyl side chain [21,22]. The above data thus demonstrated that compound **1** was a Δ^5^-3β-hydroxyl 7-oxysterol derivative, similar to the ring system of decortinone (Table 1), a known sterol previously obtained from the green alga *Codium decorticatum* [23], and the side chain of 5,28-stigmastadiene-3β,24-diol (Table 1), a known sterol previously isolated from the brown alga *Sargassum fusiforme* [24]. The stereochemistry of compound **1** at C-24 was established to be R, since the signals attributed to H-27 at δ 0.890 (3H, d, *J* = 8.0 Hz) were close to those of H-26 at δ 0.871 (3H, d, *J* = 7.2 Hz) in the ^1^H NMR spectrum of compound **1**. The chemical shift difference between H-27 and H-26 of compound **1** was 0.019 ppm, whereas the chemical shift difference between H-27 and H-21 of compound **1** was 0.041 ppm, which was in accordance with those of 24R-saringosterol. The chemical shift difference between H-27 and H-26 of 24R-saringosterol was 0.020 or 0.020 ppm, whereas the chemical shift difference between H-27 and H-21 24R-saringosterol was 0.032 or 0.033 ppm in the previous reports [21,22]. Thus, compound **1** was elucidated with the structure as shown in Figure 1, and named (24R)-5,28-stigmastadiene-3β,24-diol-7-one.

Compound **2** was isolated as a white amorphous solid. The ^13^C NMR spectrum of compound **2** displayed 29 signals (Table 1), including five quaternary carbons (one ketone carbonyl carbon, one olefinic carbon, and one oxygen-bearing carbon), nine methine carbons (two olefinic carbons and one oxygen-bearing carbon), 10 methylene carbons (one olefinic carbon), and five methyl groups. The molecular formula was established as C_29_H_46_O_3_ using HR-EI-MS (*m*/*z* 442.3439 [M]^+^, calcd. for C_29_H_46_O_3_ 442.3447). Comparison of the ^1^H and ^13^C NMR data of compound **2** with those of compound **1** showed that both compounds shared the same sterol skeleton nucleus and side chain, the only difference being that the signal of H-27 in compound **2** appeared at δ_H_ 0.91 (d, *J* = 7.2 Hz), whereas the signal of H-27 in compound **1** displayed at δ_H_ 0.89 (d, *J* = 8.0 Hz). Stereochemistry at C-24 of compound **2** was established to be *S,* since the signal attributed to H-27 at δ_H_ 0.909 (3H, d, *J* = 7.2 Hz) was very close to that of H-21 at δ_H_ 0.931 (3H, d, *J* = 6.8 Hz) in the ^1^H NMR spectrum of compound **2**. The chemical shift difference between H-27 and H-21 of compound **2** was 0.022 ppm, whereas the chemical shift difference between H-27 and H-26 of compound **2** was 0.038 ppm, which was in accordance with those of 24*S*-saringosterol. The chemical shift difference between H-27 and H-21 of 24*S*-saringosterol was 0.019 or 0.020 ppm, whereas the chemical shift difference between H-27 and H-26 24*S*-saringosterol was 0.027 or 0.027 ppm in the previous reports [21,22]. Thus, compound **2** structure was established, and named (24*S*)-5,28-stigmastadiene-3β,24-diol-7-one.

**Figure 3:**
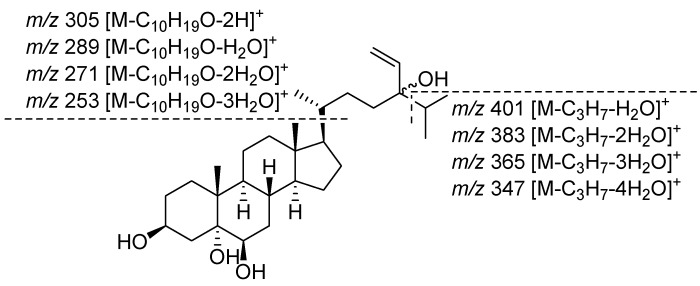
EI-MS fragments of compound **3**.

Compound **3** was isolated as a white amorphous solid and its molecular formula was assigned as C_29_H_50_O_4_ based on a pseudo-molecular ion peak at *m*/*z* 461.3625 [M − H]^−^ (calcd. for C_29_H_49_O_4_ 461.3631) in the negative HR-FAB-MS spectrum. The presence of four hydroxyl groups in compound **3** was indicated by the EI-MS spectrum, absence of the molecular ion peak, which exhibited mass ions for stepwise H_2_O loss at *m*/*z* 444 [M − H_2_O]^+^, 426 [M − 2H_2_O]^+^, 408 [M − 3H_2_O]^+^, and 390 [M − 4H_2_O]^+^. Additional prominent fragment peaks were present at *m*/*z* 401 [M − C_3_H_7_ − H_2_O]^+^, 383 [M − C_3_H_7_ − 2H_2_O]^+^, 365 [M − C_3_H_7_ − 3H_2_O]^+^, 347 [M − C_3_H_7_ − 4H_2_O]^+^, 305 [M − C_10_H_19_O − 2H]^+^, 289 [M − C_10_H_19_O − H_2_O]^+^, 271 [M − C_10_H_19_O − 2H_2_O]^+^, and 253 [M − C_10_H_19_O − 3H_2_O]^+^ (Figure 3). The ^13^C NMR spectrum of compound **3** also exhibited 29 signals (Table 1), which included five methyl groups (δ_C_ 12.1 (C-18), 16.4 (C-26), 16.8 (C-19), 17.5 (C-27), and 18.7 (C-21)), a sp^2^ methylene carbon (δ_C_ 112.7/112.6 (C-29)), two oxygenated sp^3^ methine carbons (δ_C_ 67.5 (d, C-3) and 75.9 (d, C-6)), a sp^2^ methine carbon (δ_C_ 142.2/142.1 (C-28)), two oxygenated sp^3^ quaternary carbons (δ_C_ 75.9 (C-5) and 77.5 (C-24)). The ^1^H NMR spectrum of compound **3** showed tertiary methyl groups at δ 0.67 (3H, s, H-18) and 1.18 (3H, s, H-19), and three secondary methyl groups at δ 0.87 (3H, d, *J* = 7.2 Hz, H-26), 0. 90 (d, *J* = 6.8 Hz, H-27)/0.89 (d, *J* = 8.0 Hz, H-27), and 0.914 (d, *J* = 6.4 Hz, H-21)/0.908 (d, *J* = 7.2 Hz, H-21). The 3β, 5α, 6β-trihydroxyl sterol nature was characterized by a multiplet at δ 4.10 (1H, H-3), a double doublet at δ 2.08 (1H, *J* = 12.5, 12.5 Hz, H-4), a multiplet at δ 1.60 (1H, overlap, H-4), and a broad singlet at δ 3.49 (1H, H-6), while the angular methyl groups at C-19 and C-18 resonated at δ 1.18 (s) and 0.67 (s), respectively [26]. The signal for H-6α appeared at δ 3.49 (br s), whereas the signal of H-6β resonated at about δ 3.74 (dd, *J* = 11.5, 4.5 Hz); coupling constants of H-6α in compound **3** indicated the proton to be equatorial. A singlet for H-19 was observed at δ 1.17 (s), which was shifted downfield with respect to the corresponding signal in the 6α-hydroxyl nature that resonated at δ 1.05 (s). The assignments were support by the ^13^C NMR spectral data of compound **3** and comparison with reference compounds. Up-field shifts were exhibited by C-5 at δ 75.9 and C-7 at δ 34.5, whereas the signal of C-5 and C-7 in 6α-hydroxyl nature resonated at about δ 77.0 and 38.1, respectively; downfield shifts were exhibited by δ C-6 at 75.9 and C-19 at δ 16.8, whereas the signals of C-6 and C-19 in the 6α-hydroxyl nature resonated at about δ 67.0 and 15.4, respectively [25,27]. The above data thus demonstrate that compound **3** was a 3β,5α,6β-trihydroxyl sterol derivative, similar to the ring system of (24*S*)-24-ethylcholesta-3β,5α,6β-triol, a known sterol previously isolated from the marine sponge *Spirastrella inconstans* [25]. The ^1^H NMR spectrum further exhibited an ABX system at δ 5.81 (dd, *J* = 17.2, 10.8, H-28)/5.79 (dd, *J* = 17.6, 10.8, H-28), 5.19 (dd, *J* = 17.6, 1.6 Hz, H-29)/5.18 (dd, *J* = 17.2, 2.4 Hz, H-29), and 5.14 (dd, *J* = 10.8, 2.4 Hz, H-29)/5.13 (dd, *J* = 10.8, 1.6 Hz, H-29), due to the presence of a vinyl group attached to a tertiary carbon. These data, in combination with the mass fragments, suggested that compound **3** possessed a 24-hydroxy-24-vinyl side chain and indicated that compound **3** probably consisted of epimers with the ratio of 1:1. In the HMBC spectrum of compound **3**, long-range correlations belonging to rings A and B were observed for H_3_-19 with C-1, C-5, C-9, and C-10, H-4 with C-2, C-3, C-5, C-6, and C-10, H-6 with C-4, C-5, C-9, and C-10, long-range correlations belonging to rings C and D were observed for H_3_-18 with C-12, C-13, C-14, and C-17, long-range corrections belonging to the side chain were observed for H_3_-21 with C-17, C-20, and C-22, H_3_-26 with C-24, C-25, and C-27, H_3_-27 with C-24, C-25, and C-26, H-28 with C-24, H_2_-29 with C-24 and C-28. Thus, the structure of compound **3** was determined, and it was named 24*R* and 24*S*-vinylcholesta-3β,5α,6β,24-tetraol.

Compounds **4–6** were identified by comparing the ^1^H- and ^13^C-NMR, as well as the MS spectra with those reported in the literature. They were identified as isofucoterol (**4**) [28], 24*R*,28*S* and 24*S*,28*R*-epoxy-24-ethylcholesterol (**5**) [29], and (24*S*)-5,28-stigmastadiene-3β,24-diol (**6**) [24].

Aldose reductase, an enzyme of the aldoketo reductase super-family that catalyzes the conversion of glucose to sorbitol in the polyol pathway of glucose metabolism, has been proved as the molecular target for major complications of diabetes, such as cataract, neuropathy, retinopathy, and nephropathy [30]. In this context, aldose reductase inhibitors have received much attention worldwide. In this study, compounds **1**–**6** and 15 previously isolated compounds were evaluated for human recombinant aldose reductase inhibitory activity. Quercetin, a well-known aldose reductase inhibitor [31,32,33], was used as a positive control. At the concentration of 3 μg/mL (Table 2), 24*R*,28*S* and 24*S*,28*R*-epoxy-24-ethylcholesterol (**5**), 1-*O*-palmitoyl-3-*O*-(6′-sulfo-α-d-quinovopyranosyl) glycerol, (2*S*)-1-*O*-palmitoyl-3-*O*-[α-d-galactopyranosyl(1→2)β-d-galactopyranosyl] glycerol, 4-hydroxybenzoic acid, 4-hydroxyphenylacetic acid, and 8-hydroxy-(6*E*)-octenoic acid showed weakly inhibitory activities, with inhibition values of 31.28 ± 1.04%, 27.41 ± 1.11%, 33. 89 ± 1.03%, 27.80 ± 0.79%, 33.05 ± 1.32%, and 28.92 ± 0.53%, respectively, which compared with the positive control (71.66 ± 0.52%). In addition, three new sterols **1**–**3** were almost inactive.

## 3. Materials and Methods

### 3.1. General Experimental Procedures

EIMS and FABMS were obtained using a JEOL JMS-700 mass spectrometer. NMR spectra were measured on JEOL AL-400 spectrometer (Japan Electronic Optics Laboratory Co. Ltd., Tokyo, Japan). All chemical shifts (δ) were given in ppm and the samples were solubilized in CDCl_3_ (Cambridge Isotope Laboratories, Inc., Andover, MA, USA). Optical rotations were measured by using a JASCO P-1020 automatic digital polarimeter (JASCO Corporation, Tokyo, Japan). HPLC was performed on an NPL-500 pump (Nihon Seimitsu Kagaku Co., Ltd., Tokyo, Japan) and a RI-102 detector (Showa Denko Co., Ltd., Tokyo, Japan) using a COSMOSIL Silica 5SL-II Waters column (20 mm × 250 mm, Nacalai Tesque, Inc., Kyoto, Japan) and a Senshu Pak DOCOSIL column (10 mm × 250 mm, Senshu Scientific Co. Ltd., Tokyo, Japan). Open column chromatography was performed with silica gel 60 N (100–210 μm, Kanto chemical Co., Inc., Tokyo, Japan), RP-18 reverse-phase silica gel (PEGASIL PREP ODS-5015-12A, Senshu Scientific Co. Ltd., Tokyo, Japan), and Sephadex LH-20 (Pharmacia, New Orleans, LA, USA). TLC was carried out on pre-coated TLC plates with silica gel 60 F_254_ and silica gel RP-18 60 F_254_ (0.25 mm, Merck, Darmstadt, Germany). Detection was achieved by spraying with 10% H_2_SO_4_ in MeOH and heating at 110 °C. Aldose reductase recombinant from human muscle cell purchased from Wako Pure Chemical Industries, Ltd. (Osaka, Japan). β-Nicotinamide adenine dinucleotide phosphate tetrasodium salt (NADPH), dl-glyceraldehyde, sodium dihydrogenphosphate dihydrate, disodium hydrogenphosphate 12-water, and dimethyl sulfoxide (DMSO) were bought from Nacalai Tesque, Inc. (Kyoto, Japan). Compounds isophitol [34], indole-3-carboxylic acid [35], 1-*O*-palmitoyl-3-*O*-(6′-sulfo-α-d-quinovopyranosyl) glycerol [36], (2*S*)-1-*O*-palmitoyl-3-*O*-[α-d-galactopyranosyl(1→2)β-d-galactopyranosyl] glycerol [37], 3-methylsulfoxypropionic acid [38], tyrosol [39], 4-hydroxybenzoic acid [40], loliolide [41], annuionone D [42], azelaic acid [43], succinic acid [44], 8-hydroxy-(6*E*)-octenoic acid [45], *n*-butyl β-d-fructopyranoside [46], and *n*-butyl pyroglutamate [47] were isolated and identified from this alga in our laboratory.

### 3.2. Algal Material

The wild green alga *Ulva australis* was collected at the coast of Dalian, China, in October 2002 and identified by Professor Zi’ang Yao (School of Life Science and Technology, Dalian University, Dalian, China). A voucher specimen (20021001) was deposited at the Department of Natural Medicine and Pharmacognosy, School of Pharmacy, Qingdao University, Qingdao, China.

### 3.3. Extraction and Isolation

Air-dried *U. australis* (27 kg) was powered and reflux extracted twice with 95% EtOH for 4 h. Evaporation of the solvent under reduced pressure gave the EtOH extract (1.8 kg). The EtOH extract (1.2 kg) was suspended in water and successively partitioned with hexane, EtOAc, and *n*-BuOH to give the hexane soluble part (137.9 g), EtOAc soluble part (25.7 g), and *n*-BuOH soluble part (20.2 g), respectively. The hexane soluble part was chromatographed on a silica gel column eluting with a gradient of hexane-EtOAc (20:1, 10:1, 5:1; 1:1, 1:3, *v*/*v*), EtOAc, a gradient of EtOAc-MeOH (5:1, 1:1, *v*/*v*), and MeOH to give 24 fractions (Fr. 1–Fr. 24) on the basic of TLC analyses. Fr. 10 was purified by normal-phase preparative HPLC using hexane:EtOAc (3:1, *v*/*v*) as the mobile phase and reversed-phase preparative HPLC using MeOH as the mobile phase at a flow rate of 2.0 mL/min resulting in the isolation of compounds **4** (132 mg), **5** (40.2 mg), and **6** (3.0 mg). Fr. 13 was subjected to column chromatography on Sephadex LH-20 using MeOH as an eluent, yielding sub-fractions (Fr. 13-1–Fr. 13-3). Fr. 13-2 was further separated by RP-18 silica gel column chromatography eluting with MeOH to finish sub-fractions, which were purified by reversed-phase preparative HPLC employing MeOH:H_2_O (85:15, *v*/*v*) as the mobile phase at a flow rate of 2.0 mL/min to give compound **1** (4.3 mg). Fr. 14 was isolated by Sephadex LH-20 column chromatography eluting with MeOH, RP-18 silica gel column chromatography eluting with MeOH:H_2_O (80:20, *v*/*v*), reverse-phase preparative HPLC eluting with MeOH:H_2_O (85:15, *v*/*v*) at a flow rate of 2.0 mL/min, and normal-phase preparative HPLC eluting with hexane: EtOAc (2:3, *v*/*v*) at a flow rate of 2.0 mL/min to yield compound **2** (5.5 mg). Fr. 16 was chromatographed on a RP-18 silica gel column and eluted using MeOH, and reverse-phase preparative HPLC (MeOH-H_2_O, 85:15, *v*/*v,* 2.0 mL/min) to obtain compound **3** (2.4 mg).

Compound **1**: white amorphous solid; [α]^D^_25_ −21.8 (0.02, CHCl_3_), HR-EI-MS *m*/*z* 442.3445 (calcd. for C_29_H_48_O_3_, 442.3447); EI-MS *m*/*z* 442 [M]^+^ (32), 424 (11), 399 (92), 381 (100), 344 (70), 329 (21), 285 (92); ^1^H NMR (CDCl_3_, 400 MHz) δ 5.81 (1H, dd, *J* = 17.6, 10.8, H-28), 5.69 (1H, d, *J* = 1.6 Hz, H-6), 5.20 (1H, dd, *J* = 17.6, 1.6 Hz, H-29), 5.14 (1H, dd, *J* = 10.8, 1.6 Hz, H-29), 3.67 (1H, m, H-3), 1.20 (3H, s, H-19), 0.931 (d, *J* = 6.4 Hz, H-21), 0.890 (3H, d, *J* = 8.0 Hz, H-27), 0.871 (3H, d, *J* = 7.2 Hz, H-26), 0.68 (3H, s, H-18); ^13^C NMR (CDCl_3_, 100 MHz) spectra data, see Table 1.

Compound **2**: white amorphous solid; [α]^D^_25_ −42.6 (0.01, CHCl_3_), HR-EI-MS *m*/*z* 442.3439 (calcd. for C_29_H_46_O_3_ 442.3447); EI-MS *m*/*z* 442 [M]^+^ (35), 424 (16), 399 (20), 381 (25), 344 (18), 329 (9), 285 (100); ^1^H NMR (CDCl_3_, 400 MHz) δ 5.81 (1H, dd, *J* = 17.6, 10.8, H-28), 5.69 (1H, d, *J* = 1.6 Hz, H-6), 5.19 (1H, dd, *J* = 17.6, 1.6 Hz, H-29), 5.14 (1H, dd, *J* = 10.8, 1.6 Hz, H-29), 3.67 (1H, m, H-3), 1.20 (3H, s, H-19), 0.931 (d, *J* = 6.8 Hz, H-21), 0.909 (3H, d, *J* = 7.2 Hz, H-27), 0.871 (3H, d, *J* = 7.2 Hz, H-26), 0.68 (3H, s, H-18); ^13^C NMR (CDCl_3_, 100 MHz) spectra data, see Table 1.

Compound **3** (a mixture of 24*R* and 24*S*): white amorphous solid; negative HR-FAB-MS *m*/*z* 461.3625 (calcd. for C_29_H_49_O_4_ 461.3631); EI-MS *m*/*z* 444 (11), 426 (26), 408 (8), 401 (73), 390 (2), 383 (100), 365 (68), 347 (23), 305 (83), 289 (52), 271 (70), 253 (27); ^1^H NMR (CDCl_3_, 400 MHz) δ 5.81 (dd, *J* = 17.2, 10.8, H-28)/5.79 (dd, *J* = 17.6, 10.8, H-28), 5.19 (dd, *J* = 17.6, 1.6 Hz, H-29)/5.18 (dd, *J* = 17.2, 2.4 Hz, H-29), 5.14 (dd, *J* = 10.8, 2.4 Hz, H-29)/5.13 (dd, *J* = 10.8, 1.6 Hz, H-29), 4.10 (1H, m, H-3), 3.49 (1H, br s, H-6), 2.08 (1H, *J* = 12.5, 12.5 Hz, H-4), 1.60 (1H, overlap, H-4), 1.18 (3H, s, H-19), 0.914 (d, *J* = 6.4 Hz, H-21)/0.908 (d, *J* = 7.2 Hz, H-21), 0.90 (d, *J* = 6.8, H-27)/0.89 (d, *J* = 8.0 Hz), 0.87 (3H, d, *J* = 7.2 Hz, H-26), 0.67 (3H, s, H-18); ^13^C NMR (CDCl_3_, 100 MHz) spectra data, see Table 1.

### 3.4. Human Recombinant Aldose Reductase Inhibitory Activity Assay

The human recombinant aldose reductase inhibition assays were performed according to the method modified by our group [19]. The assay was performed at 25 °C in a 200 mM sodium phosphate buffer solution (pH 6.2) (700 μL), containing 1.5 mM NADPH solution (100 μL), 100 mM dl-glyceraldehyde solution (100 μL), and 3 × 10^−2^ unit/mL human recombinant aldose reductase solution (100 μL), in the total volume of 1.0 mL. The effects of each sample on the enzyme activity were determined by adding 3 μL of test sample solution (final concentration 3 μg/mL dissolved in 100% DMSO) to the reaction mixture. An appropriate blank control mixture and positive control mixture were prepared. The reaction was initiated by the addition of dl-glyceraldehyde solution and the rate of NADPH oxidation was followed by recording the decrease in the absorbance at 340 nm on a UV spectrophotometer (SHIMADZU UV 1600, Kyoto, Japan). Inhibition percentage (%) was calculated as [1 − (∆A_s_ − ∆A_b_)/(∆A_c_ − ∆A_b_)] × 100, where A_s_ is the decreased absorbance of the sample, A_c_ and A_b_ are the decreased absorbances without a sample as a positive control and without a sample and enzyme as a blank control, respectively. An overview about the effects of these substances on human recombinant aldose reductase inhibitory activity is given in Table 2.

## 4. Conclusions

Phytochemistry investigation of the green alga *U*. *australis* led to the isolation of three new sterols and three known sterols. These compounds, together with the previously isolated secondary metabolites of this alga, were investigated for their inhibitory effects on aldose reductase in vitro. Of the compounds, 24*R*,28*S* and 24*S*,28*R*-epoxy-24-ethylcholesterol (**5**), 1-*O*-palmitoyl-3-*O*-(6′-sulfo-α-d-quinovopyranosyl) glycerol, (2*S*)-1-*O*-palmitoyl-3-*O*-[α-d-galactopyranosyl(1→2)β-d-galactopyranosyl] glycerol, 4-hydroxybenzoic acid, 4-hydroxyphenylacetic acid, and 8-hydroxy-(6*E*)-octenoic acid weakly inhibited the enzyme. The obtained results are beneficial to subsequent research on this alga.

## Figures and Tables

**Table 1:** ^13^C-NMR spectral data of compound **1**–**3** (100 MHz, CDCl_3_, δ in ppm).

Position	1	2	3	a ^1^	b ^2^	c ^3^	d ^4^
1	36.3	36.3	30.8	36.3	37.3	37.3	30.2
2	31.1	31.2	32.3	31.1	32.0	32.0	33.3
3	70.4	70.5	67.5	70.4	71.8	71.8	66.8
4	41.7	41.8	40.7	41.7	42.4	42.4	39.7
5	164.6	164.8	75.9	164.9	140.7	140.7	75.0
6	125.8	126.0	75.9	126.1	121.6	121.6	75.4
7	201.6	201.9	34.5	202.5	31.7	31.7	35.4
8	45.3	45.4	30.1	45.3	32.0	32.0	30.0
9	49.8	49.9	45.8	49.9	50.2	50.2	45.0
10	38.2	38.2	38.2	38.6	36.6	36.6	37.7
11	21.2	21.2	21.2	21.1	21.2	21.1	20.8
12	38.6	38.6	39.8	38.6	39.8	39.8	39.1
13	43.0	43.1	42.7	43.0	42.4	42.3	42.3
14	49.8	49.9	55.8	49.8	56.8	56.8	55.9
15	26.2	26.2	24.0	26.2	24.3	24.3	22.2
16	29.6	29.7	28.14/28.09	29.3	28.2	28.3	27.6
17	54.4	54.5	55.8	54.6	55.9	55.9	55.6
18	11.9	12.0	12.1	11.6	11.9	11.9	11.6
19	17.3	17.3	16.8	17.2	19.5	19.4	16.1
20	36.0	36.0	36.0	35.4	36.0	36.0	35.8
21	18.9	18.9	18.7	18.7	18.9	18.8	18.2
22	29.0	29.1	29.0	33.3	29.1	29.2	33.6
23	34.8	34.8	34.8/34.6	29.6	34.8	34.6	23.7
24	77.5	77.6	77.5	49.4	77.7	77.7	45.0
25	35.8	35.8	35.9	17.8	36.1	36.2	27.8
26	16.4	16.4	16.4	147.5	16.5	16.5	18.3
27	17.5	17.5	17.5	111.3	17.6	17.6	18.3
28	142.1	142.3	142.2/142.1	26.4	142.4	142.5	22.0
29	112.7	112.8	112.7/112.6	11.9	112.9	112.8	11.8

^1^ a, Decortinone, data from [23]; ^2^ b, (24*R*)-5,28-stigmastadiene-3β,24-diol, data from [24]; ^3^ c, (24*S*)-5,28-stigmastadiene-3β,24-diol, data from [24]; ^4^ d, (24*S*)-24-ethylcholesta-3β,5α,6β-triol, data from [25].

**Table 2:** Human recombinant aldose reductase inhibitory activities of the isolated compounds.

Compounds	Inhibitory Ratio (%)
1	3.31 ± 0.85
2	4.08 ± 0.39
3	2.87 ± 0.62
4	8.13 ± 1.76
5	31.28 ± 1.04
6	N.I. ^1^
Isophitol	21.86 ± 1.21
Indole-3-carboxylic acid	10.74 ± 0.92
1-*O*-Palmitoyl-3-*O*-(6′-sulfo-α-d-quinovopyranosyl) glycerol	27.41 ± 1.11
(2*S*)-1-*O*-Palmitoyl-3-*O*-[α-d-galactopyranosyl(1→2)β-d-galactopyranosyl] glycerol	33. 89 ± 1.03
3-Methylsulfoxypropionic acid	12.42 ± 0.63
Tyrosol	15.81 ± 1.16
4-Hydroxybenzoic acid	27.80 ± 0.79
4-Hydroxyphenylacetic acid	33.05 ± 1.32
Loliolide	8.46 ± 1.15
Annuionone D	18.74 ± 0.92
Azelaic acid	13.38 ± 0.59
Succinic acid	15.98 ± 0.87
8-Hydroxy-(6*E*)-octenoic acid	28.92 ± 0.53
*n*-Butyl β-d-fructopyranoside	6.41 ± 0.88
*n*-Butyl pyroglutamate	16.38 ± 1.87
Quercetin	71.66 ± 0.52

^1^ N.I. = inactive at 3 μg/mL.

## Supplementary Materials

Supplementary File 1
