# Supplementary material for: Sterols from the Green Alga Ulva australis
Source: Mar Drugs. 2017 Sep 28;15(10):299. doi: 10.3390/md15100299 (PMC5666407; doi:10.3390/md15100299)
Supplement: Supplementary File 1 [file marinedrugs-15-00299-s001.pdf]

Supplementary Material: Sterols from the Green Alga *Ulva australis*

Guo-Liang Li, Wei-Jie Guo, Guang-Bao Wang, Rong-Rong Wang, Yu-Xue Hou, Kun Liu, Yang Liu and Wei Wang

Supporting information

Spectroscopic data of compounds **4–6**

Compound **4**: white amorphous solid; $[\alpha]_D^{25}$ –36.4° (*c* 0.02, $CDCl_3$); HR-EI-MS *m*/*z* 412.3708 (calcd for $C_{29}H_{48}O$ 412.3705); EI-MS *m*/*z* 412 $[M]^+$ (11), 314 (100); ^{1}H NMR ($CDCl_3$, 400 MHz) δ 5.35 (1H, br d, *J* = 5.2 Hz, H-6), 5.11 (1H, q, *J* = 6.8 Hz, H-28), 3.53 (1H, m, H-3), 2.83 (1H, m, H-25), 1.59 (3H, d, *J* = 6.8 Hz, H-29), 1.01 (3H, s, H-19), 0.98 (6H, d, *J* = 6.8 Hz, H-26, 27), 0.95 (3H, d, *J* = 6.4 Hz, H-21), 0.68 (3H, s, H-18); ^{13}C NMR ($CDCl_3$, 100 MHz) δ 145.7 (C-24), 140.6 (C-5), 121.6 (C-6), 116.4 (C-28), 71.8 (C-3), 56.8 (C-14), 56.1 (C-17), 50.2 (C-9), 42.4 (C-13), 42.4 (C-4), 39.9 (C-12), 37.4 (C-1), 36.6 (C-10), 36.2 (C-20), 36.1 (C-22), 32.0 (C-8), 32.0 (C-7), 31.8 (C-2), 28.7 (C-25), 28.4 (C-16), 28.1 (C-23), 24.4 (C-15), 21.2 (C-11), 21.2 (C-26 or 27), 21.1(C-26 or 27), 19.5 (C-19), 19.0 (C-21), 12.9 (C-29), 12.0 (C-18).

Compound **5** (a mixture of 24*R*,28*S* and 24*S*,28*R*): white amorphous solid; HR-EI-MS *m*/*z* : 428.3658 (calcd for $C_{29}H_{48}O_2$, 428.3654); EI-MS *m*/*z* 428 $[M]^+$ (14), 410 (17), 314 (100), 271 (45); ^{1}H NMR ($CDCl_3$, 400 MHz) δ 5.35 (1H, br. d, *J* = 5.2 Hz, H-6), 3.53 (1H, m, H-3), 2.95 (1H, q, *J* = 5.2 Hz, H-28), 1.29 (3H, d, *J* = 6.0 Hz, H-29), 1.06/1.05 (3H, d, *J* = 7.2 Hz, H-27), 1.01 (3H, s, H-19), 0.96 (3H, d, *J* = 7.2 Hz, H-26), 0.91 (3H, d, *J* = 6.4 Hz, H-21), 0.67 (3H, s, H-18); ^{13}C NMR ($CDCl_3$, 100 MHz) δ 140.6 (C-5), 121.6 (C-6), 71.8 (C-3), 66.2 (C-24), 58.0/57.6 (C-28), 56.8 (C-14), 56.0/55.8 (C-17), 50.2 (C-9), 42.4 (C-13), 42.4 (C-4), 39.8 (C-12), 37.4 (C-1), 36.6 (C-10), 36.2/36.1 (C-20), 32.0 (C-8), 32.0 (C-7), 31.8 (C-2), 31.0 (C-25), 30.5/30.2 (C-22), 28.3 (C-16), 25.9/25.5 (C-23), 24.4 (C-15), 21.2 (C-11), 19.5 (C-19), 19.01 (C-21), 18.8 (C-27), 18.0/17.9 (C-26), 13.6/13.6 (C-29), 12.0 (C-18).

Compound **6**: colorless crystal; HR-EI-MS *m*/*z* : 428.3659 (calcd for $C_{29}H_{48}O_2$, 428.3654); EIMS *m*/*z* 428 $[M]^+$ (17), 410 (31), 385 (34), 367 (100), 328 (6), 314 (25), 271 (97);^{1}H NMR ($CDCl_3$, 400 MHz) δ 5.79 (1H, dd, *J* = 17.6, 10.8, H-28), 5.35 (1H, br d, *J* = 5.2 Hz, H-6), 5.18 (1H, dd, *J* = 17.6, 1.6 Hz, H-29), 5.127 (1H, dd, *J* = 10.8, 1.2 Hz, H-29), 3.52 (1H, m, H-3), 1.00 (3H, s, H-19), 0.92 (d, *J* = 7.6 Hz, H-21), 0.90 (3H, d, *J* = 7.2 Hz, H-27), 0.87 (3H, d, *J* = 7.2 Hz, H-26), 0.67 (3H, s, H-18); ^{13}C NMR ($CDCl_3$, 100 MHz) δ 142.4 (C-28), 140.6 (C-5), 121.5 (C-6), 112.7 (C-29), 77.6 (C-24), 71.7 (C-3), 56.7 (C-14), 55.8 (C-17), 50.1 (C-9), 42.3 (C-4), 42.3 (C-13), 39.7 (C-12), 37.2 (C-1), 36.5 (C-10), 36.1 (C-25), 35.9 (C-20), 34.5 (C-23), 31.9 (C-2, 8), 31.6 (C-7), 29.1 (C-22), 28.2 (C-16), 24.2 (C-15), 21.1 (C-11), 19.4 (C-19), 18.8 (C-21), 17.5 (C-27), 16.4 (C-26), 11.8 (C-18).
